# OCT angiography in the monitoring of vaginal health

**DOI:** 10.1063/5.0153461

**Published:** 2023-11-07

**Authors:** Saijun Qiu, Afiba Arthur, Yuchen Jiang, Yusi Miao, Yan Li, Jingyi Wang, Yona Tadir, Felicia Lane, Zhongping Chen

**Affiliations:** 1 Beckman Laser Institute, University of California Irvine, Irvine, California 92612, USA; 2 Department of Biomedical Engineering, University of California Irvine, Irvine, California 92612, USA; 3 Department of Obstetrics and Gynecology, University of California Irvine Medical Center, Orange, California 92868, USA; 4 Department of Electrical Engineering and Computer Science, University of California Irvine, Irvine, California 92612, USA

## Abstract

Fractional-pixel CO_2_ laser therapy shows promise for treating the genitourinary syndrome of menopause (GSM). Nevertheless, it remains controversial in the field of female pelvic medicine. This is due to the inherent difficulties in obtaining noninvasive biopsies to evaluate the treatment's efficacy and safety objectively. To address this challenge, we developed a noninvasive intravaginal optical coherence tomography (OCT)/OCT angiography (OCTA) endoscopic system, whose probe features a shape identical to the laser treatment probe. This system can provide high-resolution OCT images to identify the microstructure of vaginal tissue and visualize the vasculature network *in vivo*. We conducted clinical research on 25 post-menopausal patients with GSM. OCT/OCTA scans were acquired at four different locations of the vagina (distal anterior, distal posterior, proximal anterior, and proximal posterior) during the whole laser treatment session. A U-Net deep learning model was applied to segment the vaginal epithelium for assessing vaginal epithelial thickness (VET). Blood vessel density and VET were quantified to monitor the efficacy of fractional-pixel CO_2_ laser therapy. Statistical correlation analyses between these metrics and other clinical scores were conducted, validating the utility of our system. This OCT/OCTA endoscopic system has great potential to serve as a noninvasive biopsy tool in gynecological studies to screen, evaluate, and guide laser treatment for GSM.

## INTRODUCTION

Genitourinary Syndrome of Menopause (GSM) is a prevalent condition affecting a significant proportion of post-menopausal women, with symptoms such as vaginal dryness, burning, irritation, dysuria, urgency, and recurrent urinary tract infections (UTIs).^1–3^ These symptoms are primarily a result of a physiologic drop in estrogen production, which leads to various changes in the genitourinary system. GSM affects between 27% and 84% of post-menopausal women.^4–7^ In addition, 19% of women in the age group 40–45 recently reported that this syndrome has occurred in the pre-menopausal years.^8–14^ Since GSM has a substantial impact on the quality of life of affected women, it is critical to warrant effective treatment options for women.

Hormone therapy is currently the only approved method in the market that relieves the GSM symptoms by supplementing the body's natural estrogen directly. However, there are safety risks related to prolonged hormone therapy, such as an increased risk of breast cancer, blood clots, and endometrial cancer.^15^ Von Schoultz *et al.* proposed that different doses of estrogen and progesterone for menopausal hormone treatment may be associated with breast cancer recurrence.^16^ Thus, hormone therapy cannot cover every patient's needs. There is still a need for alternative treatments to address this common clinical issue. Recently, energy-based therapy (EBT) was introduced into the gynecological field, such as fractional-pixel CO_2_ laser, which could induce collagen formation, angiogenesis, and epithelial thickness thickening to improve vaginal tissue health by causing microtrauma. However, U.S. Food and Drug Administration (FDA) issued a communication warning against the marketing of EBT devices for specific indication in 2018 because of a lack of evidence for their effectiveness and safety, which impedes the implementation.

A significant challenge in assessing the effectiveness and safety of GSM treatments, including EBT, is the difficulty in objectively evaluating treatment outcomes. Current evaluation methods primarily rely on subjective indices from clinicians and patients without any objective physiological evidence to support their conclusions.^17^ Clinicians typically use a scoring system known as the Vaginal Health Index (VHI) to assess vaginal health based on factors of vaginal elasticity, fluid volume, pH, epithelial integrity, and moisture on a scale of 1–5.^18^ While this scoring system provides some insight into the patient's condition, it is vulnerable to inter- and intra-observer variation, limiting it as a reliable assessment tool. On the other hand, patients may complete questionnaires such as the Vulvovaginal Symptom Questionnaire (VSQ), which has demonstrated some utility in assessing the severity of GSM symptoms. However, as both VHI and VSQ are subjectively derived from clinicians and patients, respectively, these scores do not always correlate well with each other, further highlighting the need for a more objective assessment method. An additional challenge in evaluating GSM treatment outcomes is the unwillingness of patients to undergo invasive biopsy examinations, which could provide more definitive information about the anatomical changes associated with menopause and its treatments. In the absence of clinical justification for performing a biopsy for GSM, current published data lack robust, objective information about the effectiveness of various treatment options. Thus, there is a significant need for a noninvasive tool that can acquire objective anatomical information to assess vaginal health and determine treatment efficacy.

Given that estrogen deficiency leads to reduced vascularization and thinning of epithelial thickness in the vagina, several approaches have attempted to evaluate the condition of blood vessels and epithelial thickness in the vagina noninvasively.^19^ However, these methods have had varying degrees of limitations. For example, transvaginal ultrasound (TVUS), which can be used to evaluate macrostructures such as the uterus and ovaries, cannot precisely measure microstructures like epithelium.^20^ This is because the spatial resolution of ultrasound is 50–200 *μ*m, while the vaginal epithelial thickness (VET) of GSM patients is typically about 100–200 *μ*m.^21^ In comparison, optical coherence tomography (OCT) offers a much higher resolution that can achieve measurements on the order of several micrometers. Our group has previously published work on 3D scanning of *in vivo* human vaginal canals using a rotated OCT imaging probe to measure the epithelial thickness.^22^ Furthermore, we conducted a pilot study demonstrating the feasibility of using OCT to provide an optical biopsy of the vaginal lamina propria and epithelium.^23^ However, in addition to vaginal tissue structure change, vaginal vascular change is also an important indication of vagina health. Although photoplethysmography (PPG) can assess blood flow in vaginal tissue, it cannot visualize the vascular networks in the vaginal wall.^24^

In this paper, we report the development of an endoscopic OCT/OCTA system that enables noninvasive imaging of vaginal microvasculature with high spatial resolution and high imaging speed. Our novel imaging system is integrated with a clinical treatment laser by sharing an identical outer probe sleeve, providing a smooth transition between diagnosis and treatment while minimizing the risk of infection. We present the first clinical studies of vaginal vasculature in patients with GSM using the developed OCT/OCTA system. 25 patients were enrolled in these studies, and OCT/OCTA scans were acquired throughout the treatment session, including three laser treatments with four- to six-week intervals. To assess the effect of fractional-pixel CO_2_ laser therapy, we performed statistical analyses of blood vessel density (BVD) and vaginal epithelial thickness (VET) data obtained from OCT/OCTA images. Pearson's correlation coefficients were calculated to validate the relationship between the measured parameters and the subjective scores derived from the VHI and VSQ. Our results demonstrate that this OCT/OCTA system enables quantitative analysis for screening and evaluating GSM treatment. The OCT/OCTA system potentially serves as a noninvasive biopsy tool in gynecological studies, allowing clinicians to evaluate treatment responses more comprehensively. By addressing the limitations of existing assessment methods and providing a more objective and reliable means of evaluating GSM treatment outcomes, our OCT/OCTA system has the prospect to facilitate the development and approval of alternative treatments such as EBT for GSM, ultimately improving the quality of life for affected women.

## RESULTS

**FIG. 1.**
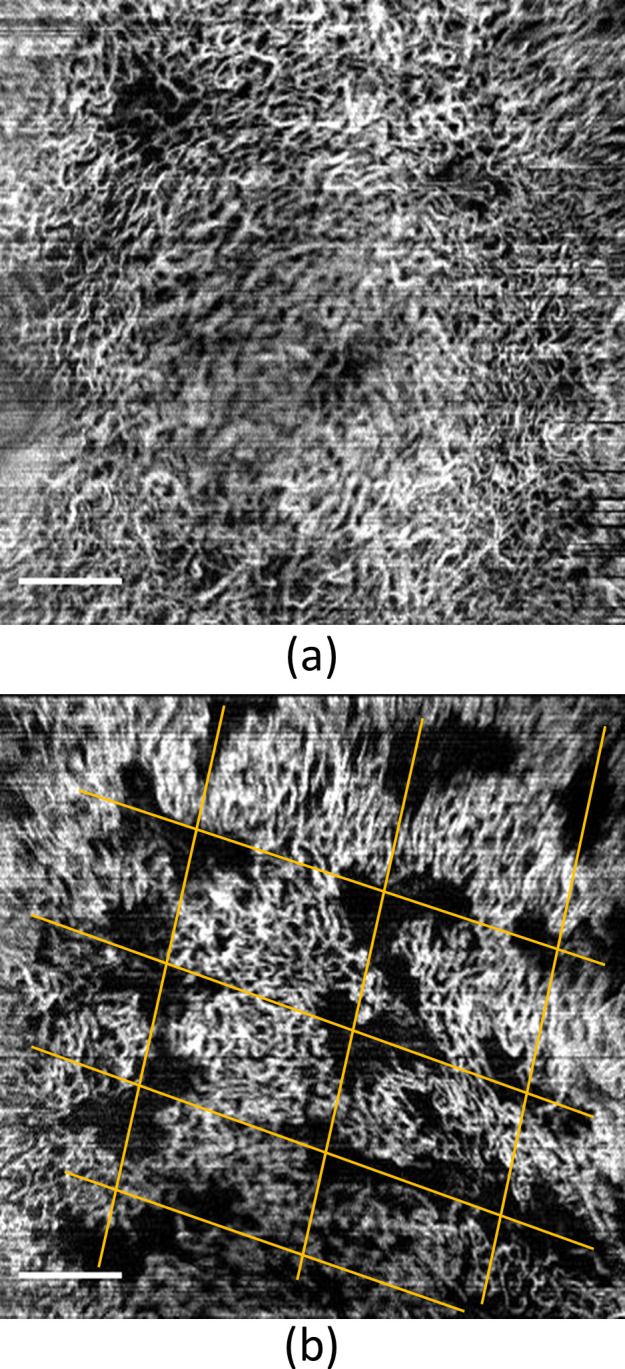
*En* *face* OCTA at proximal posterior vagina. (a) Before the treatment. (b) After the treatment. The yellow lines mark the grid-like treatment pattern. Scale bar: 1mm.

Figures 1(a) and 1(b) show the *en-face* projection views of blood vessel networks in the proximal posterior vagina from a post-menopausal patient, which are obtained before- and after-treatment during the same visit. The grid-like treatment pattern from the fractional-pixel CO_2_ laser is evident in Fig. 1(b). These results confirm that our endoscopic OCTA system is capable of capturing changes in the vascular network before and after the treatment, illustrating its potential for monitoring treatment progress.

**FIG. 2.**
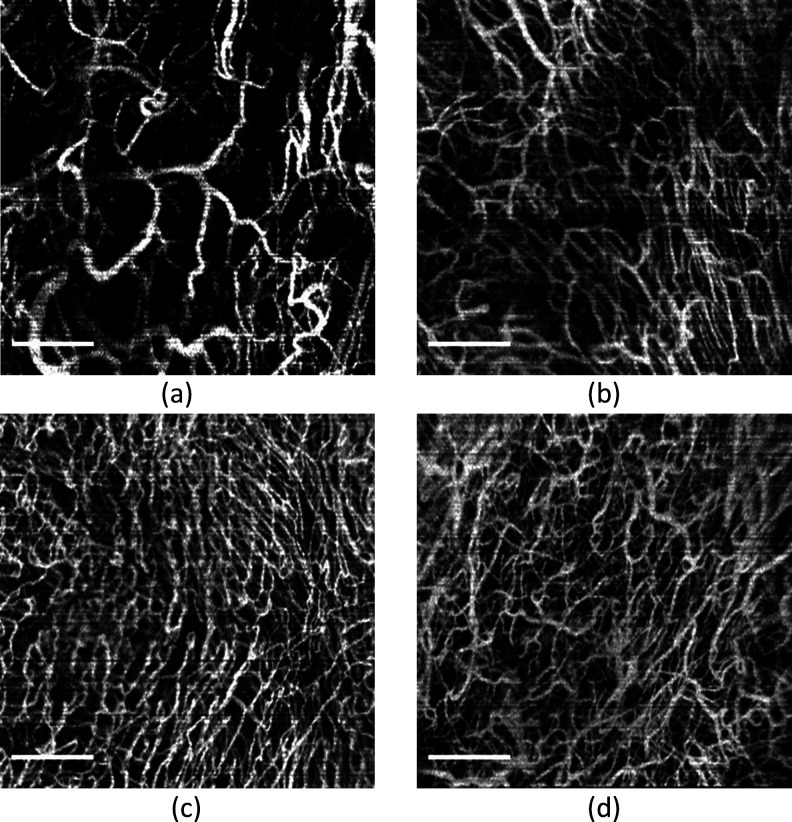
The series of OCTA images of the same location from the first to fourth visit. (a) First visit. (b) Second visit. (c) Third visit. (d) Fourth visit. Scale bar: 1 mm.

Figure 2 presents a series of OCTA images from four visits at the same location for another patient. The BVD values are 25.1% (a), 37.8% (b), 40.8% (c), and 41.1% (d), respectively, demonstrating that the vasculature becomes dense after the laser treatments. This increased density is indicative of improved tissue health and suggests that laser treatments are effectively promoting angiogenesis.

**FIG. 3.**
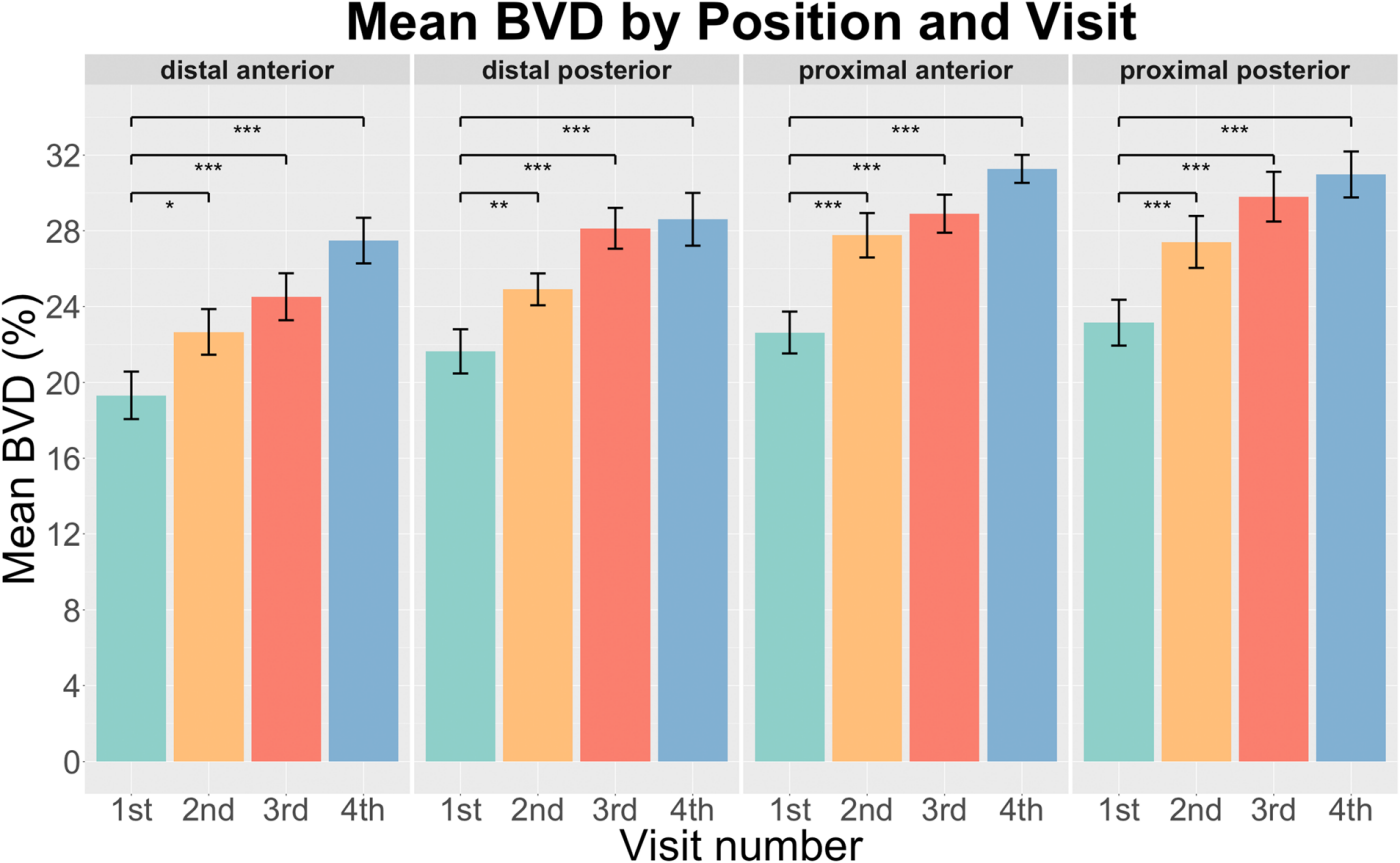
Average BVD measurements across different visits for each location (^***^p < 0.001, ^**^p < 0.01, and ^*^p < 0.05).

**FIG. 4.**
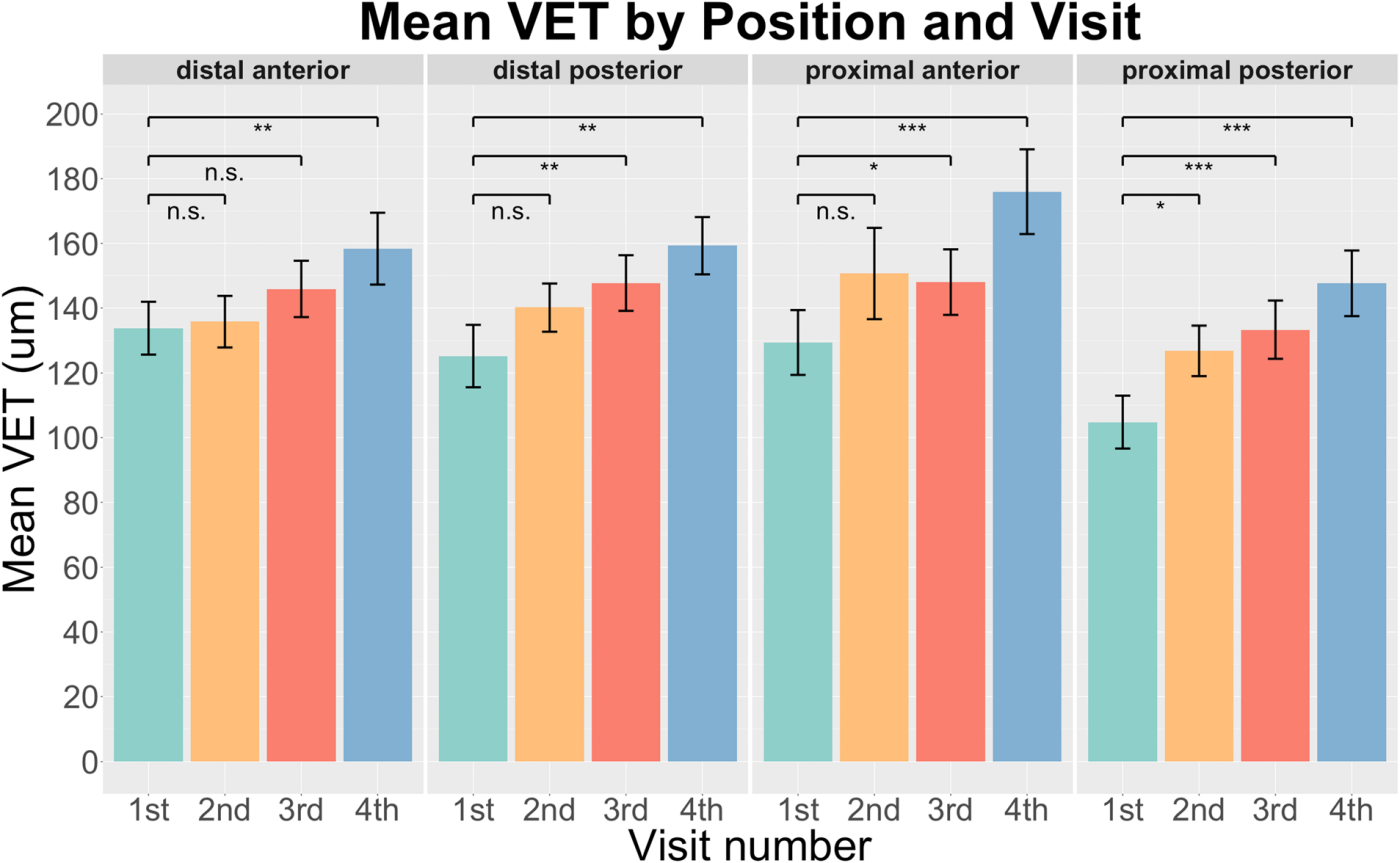
Average VET measurements across different visits for each location (^***^p < 0.001, ^**^p < 0.01, ^*^p < 0.05, and ns: not significant).

We quantified the BVD and VET at four locations (distal anterior (da), distal posterior (dp), proximal anterior (pa), and proximal posterior (pp)) from the first to the fourth measurement, offering a comprehensive view of the treatment's impact on different regions of the vaginal tissue. The mean of BVD and VET changes after the treatment at all four locations are shown in Figs. 3 and 4, respectively. A P-value less than 0.05 indicates statistical significance. All four positions showed a significant increase in BVD as the treatment went on (Fig. 3), confirming the treatment's efficacy in providing angiogenesis across the whole vaginal area. The most noticeable change was in the pa position, where the blood vessel density went up from 22.6% at the baseline to 31.3% by the fourth visit. A trend similar to that of BVD was observed for VET across the da, dp, and pp positions, with the exception of the pa location, where a slight decrease was observed after the second treatment (Fig. 4). Nevertheless, the pa location demonstrated the most notable increase across all four locations in both BVD and VET when compared to baseline metrics, which are 8.6% and 46.6 *μ*m, respectively. Although not every visit exhibited statistically significant growth compared to baseline measurements, the final visit showed substantial variation across all positions when compared with baseline. Combining data from four locations and four visits, the mean of VET increases from 123.3 μm to 160.3  *μ*m, and the mean of BVD increases from 21.7% to 29.6%. These increases demonstrate the substantial impact of the laser treatments on both blood vessel density and epithelial thickness, two critical factors in alleviating GSM symptoms and enhancing tissue functionality.

The absolute value of the Pearson's correlation coefficient between VHI and VSQ, VSQ and BVD, and VHI and BVD are 0.754, 0.310, and 0.384, respectively. The correlation between BVD and subjective scores could be categorized as moderately correlated, indicating a connection between the observed increase in blood vessel density and subjective scores from clinicians and patients. The coefficient between VHI and VET is 0.853, and the coefficient between VSQ and VET is 0.721. The coefficient value between VHI and VET is the highest among these measurements. The strong correlations between VET and subjective scores suggest that our approach has the potential to serve as a powerful tool for evaluating vaginal health from both subjective and objective scopes. By combining OCTA-derived parameters such as BVD and VET with subjective measures like VHI and VSQ, clinicians can obtain a more comprehensive understanding of a patient's vaginal health and tailor treatment strategies accordingly.

## DISCUSSION

In this study, we developed an endoscopic OCT/OCTA imaging system integrated with the laser treatment probe. To validate our system, we compared the OCTA images obtained before and after treatment during the same visit. Our system was able to capture the treatment patterns and demonstrate its capability to visualize and monitor the change in BVD across a series of OCTA images. We conducted clinical research on 25 post-menopausal patients with GSM and quantified the change in BVD and VET along with their fractional-pixel CO_2_ laser treatments, which demonstrates the reproducibility of our endoscopic OCT system in acquiring anatomic information and tracking treatment progress.

There are three key innovations in our current studies. First, to the best of our knowledge, this is the first instance where the changes in the vaginal vascular network following laser treatment have been observed and measured. The imaging results show that our system has great potential to evaluate any kind of hormonal or emerging energy-based treatments, including but not limited to laser treatment. The technical ability to evaluate histologic change at distinct vaginal sites, from the distal anterior to the mid- and proximal posterior, may offer valuable information once different treatment modalities for GSM are compared on a larger database. This is a significant advancement in the field, as it provides a noninvasive method to assess treatment efficacy in objective scientific terms. Second, we employed relay lens series to make the imaging probe share the same metal housing as the treatment probe. This unique design allows imaging and treatment phases to share the same disposable outer cover, providing a smooth transition between the diagnosis and the treatment. Third, the clinical trial with 25 enrollees shows the robustness of our system. The successful implementation of our system in a clinical setting highlights its practical applicability and serves as a foundation for future research on a larger scale. This study has the potential to be applied in extensive clinical trials related to OCT imaging in the gynecologic field.

Despite our preliminary results from 25 patients clearly indicating the power of endoscopic OCTA in assessing the change in vascular networks following treatment, challenges still remain in translating this technology to the clinic. For example, in our study, the correlation between BVD, VET, VSQ, and VHI is mostly moderate, which indicates that neither subjective scores from patients and clinicians nor objective anatomy could provide a complete gold standard for evaluating vaginal health at current stage. This gap may have two potential explanations, both of which warrant further investigation. First, the relatively small sample size of 25 patients could limit the statistical power of our analysis to achieve a more robust correlation. A future study with a larger and more diverse patient population is ongoing to further demonstrate the correlations. Second, the moderate correlation may also reflect the inherent limitations of subjective scoring methods. Subjective scores would introduce a range of biases and individual variability that are not present in objective measurements. Additionally, our findings prompt us to consider whether subjective scores alone can serve as a reliable method for understanding the histologic and functional changes, thus underscoring the importance of integrating objective tools for a more comprehensive evaluation. Moreover, the moderate correlations suggest that further research is needed to develop more comprehensive assessment tools that can accurately evaluate the effectiveness of laser treatments for GSM. To better understand GSM and assess the efficacy of the treatment methods, additional tissue properties measurement can further enhance our understanding of the energy-based treatment of GSM. In future work, OCT elastography (OCE) would also be integrated into this OCT/OCTA system to measure the mechanical property of the vagina. In addition, a large clinical trial is essential to confirm our initial findings. To support that, more efficient data analyses, including the deep learning network and regression analysis, are significant for analyzing a large number of image data. This will help to screen the patients who will most benefit from any treatment, whether it is hormonal or energy-based, while also optimizing laser duration and dose, and further individualizing treatment strategies.

In summary, we developed an endoscopic OCT/OCTA imaging system integrated with the laser treatment probe. Our pilot clinical trial demonstrated that the technology could be used to quantify BVD and VET before and after treatment. The OCT/OCTA endoscopic system demonstrated a great potential to serve as a noninvasive tool in screening, guiding, and evaluating laser treatment of GSM.

## METHODS

### System setup and endoscope design

**FIG. 5.**
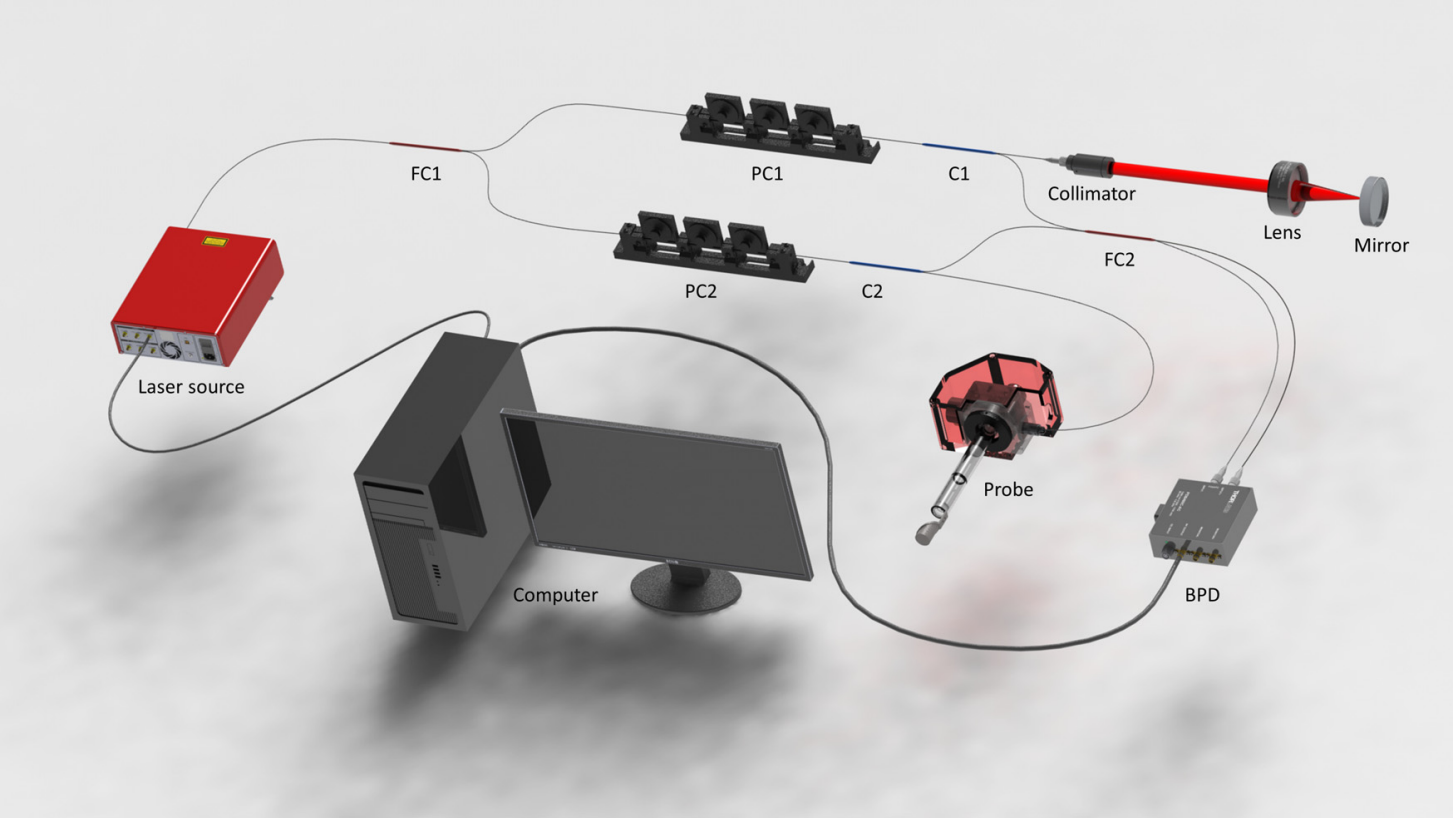
Schematic of the system. FC: fiber coupler. PC: polarization controller. C: fiber circulator. BPD: balanced photodetector.

The swept-source OCT system is illustrated in Fig. 5. A 100-kHz, 1310-nm swept-source laser (SL1310V1–10048, Thorlabs Inc.) was applied as the light source. A fiber coupler with a 90/10 split ratio was used to divide the laser beam into the sample and reference arms. Two circulators were employed in the reference and sample arms to direct the back-reflected and back-scattered light back to another fiber coupler with a 50/50 split ratio. Subsequently, the interference signal generated from the coupler is then sent to a balanced photodetector (PDB470C-AC, Thorlabs Inc.).

**FIG. 6.**
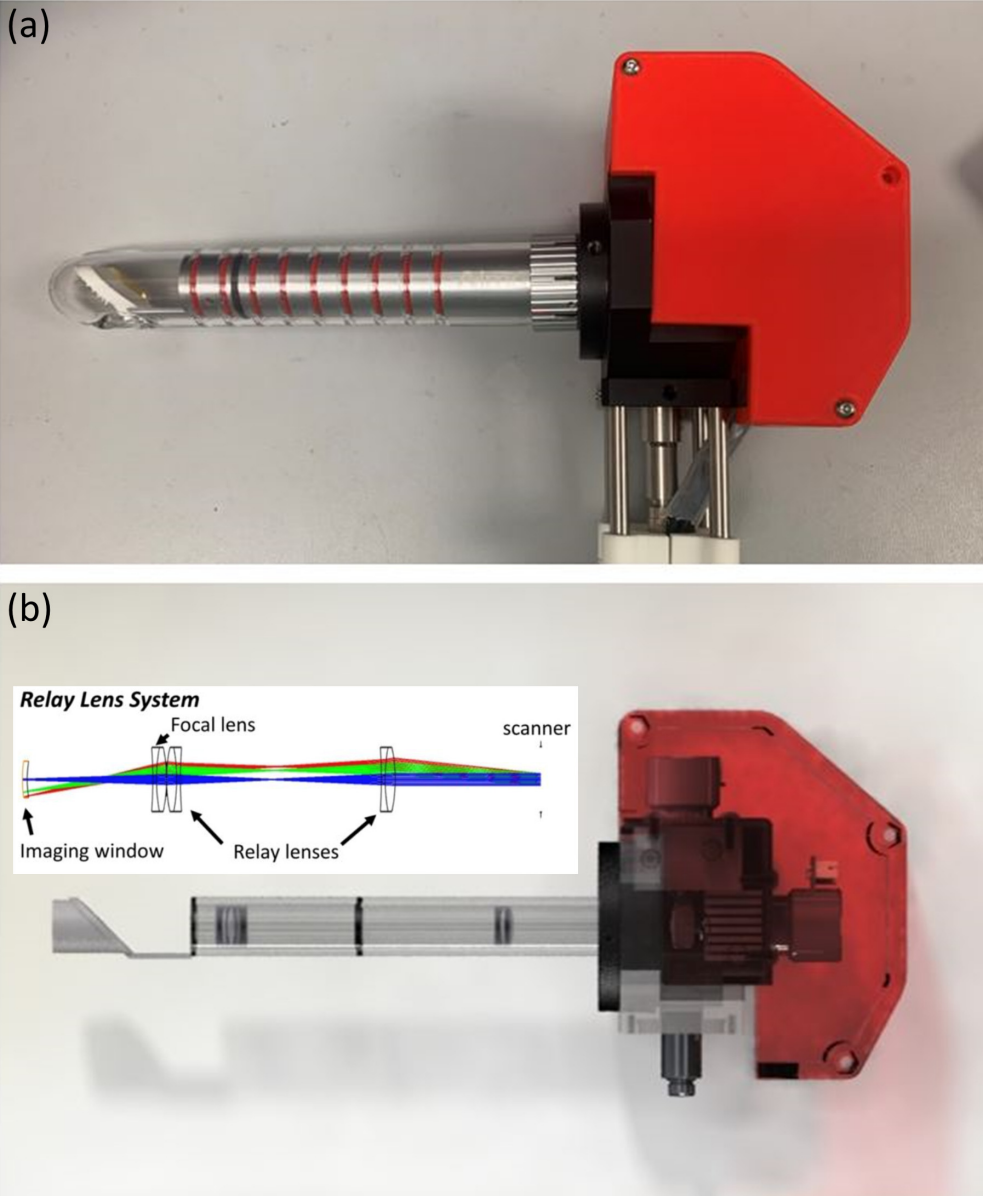
Endoscope design: (a) the picture of the probe, and (b) the SOLIDWORKS drawing of the probe.

The endoscope design is shown in Fig. 6, which consists of a collimator, a high-speed 2-axis scanner, a relay lens system, and a 45° reflective mirror. The probe shares the identical distal component as the laser ablation device employed in this study. To accommodate this feature, the length of the lens system was extended to 15 cm, matching the laser probe. Three achromatic doublets were utilized to assemble the lens system. The relay lens was placed back-to-back with the focal lens. The distance between the relay lens and the other lens, determined through Zemax simulation, controls the focal distance of 50 mm. This design also provides a large field of view (9 × 9 mm^2^). The 30-mm outer diameter of the probe enables imaging without interference from vaginal folds. The sensitivity and axial resolution of the system are 104 dB and 14 *μ*m in tissue, respectively.

### Image processing and measurement

The intensity-based Doppler variance (IBDV) algorithm was employed to extract angiography signals.^25,26^ This method evolved from the phase-resolved Doppler variance method based on an autocorrelation algorithm. The IBDV method computes the variance of the intensity differences between neighboring A-lines in consecutive B-scans, represented by the following equation:

$$\sigma^{2}=1-\frac{\sum_{j=1}^{J}\left|A_{j,z}\right|\left|A_{j+1,z}\right|}{\sum_{j=1}^{J}\left(\frac{{\left|A_{j,z}\right|}^{2}+\left|{A_{j+1,z}}^{2}\right|}{2}\right)},$$

where $\left|A_{j,z}\right|$ is the amplitude signal at *j*th A-line at a depth of z, and J is the number of averaged A-lines. Due to the longer time intervals between neighboring A-lines, the inter-frame-based IBDV method provides enhanced sensitivity for microvasculature imaging, allowing for more detailed visualization of small vessels. *En-face* OCTA images are obtained by average projecting the resliced Doppler variance images.

BVD is defined as the ratio of the vessel area with blood flow to the total area measured. A Hessian-based Frangi Vesselness filter was applied to *en-face* OCTA images, improving the visibility of blood vessel networks^27^ and enabling more accurate BVD quantification. In OCT images, the epithelium layer appears as a dark layer between the surface reflection and the bright lower layer of the lamina propria. A deep learning segmentation algorithm based on U-Net was used to extract the epithelium layer.^28^ The U-Net algorithm is a type of convolutional neural network specifically designed for biomedical image segmentation. It features a symmetric encoder–decoder architecture with skip connections, enabling precise localization and accurate segmentation of the target layer. For the empirical evaluation, a dataset of 2750 pairs of vaginal OCT images was assembled. The corpus was divided into subsets for training (2000 images), validation (500 images), and testing (250 images). Each image was manually labeled to identify the epithelium layer by an expert OCT reader. The model demonstrated exemplary performance, with threshold of 0.6, achieving a pixel accuracy of 0.989, an Intersection over Union (IoU) score of 0.836, and a Dice coefficient of 0.908.

### *In vivo* human study and experiment protocol

**FIG. 7.**
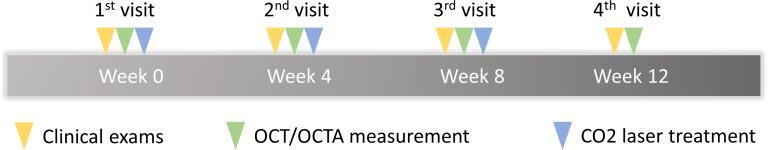
Study timeline.

25 post-menopausal women were enrolled in this study with a mean age of 62.6 (SD 8.5). They were clinically diagnosed as GSM by gynecologists. All study participants indicated the presence of vulvovaginal dryness and coital discomfort on their preliminary VSQ. The research methodology entailed a series of four appointments for each subject, occurring at intervals of four to six weeks. At these scheduled visits, participants completed the VSQ, underwent a vaginal examination by a clinician, and had their VHI recorded. Vaginal OCT measurement was conducted at each visit. The initial three visits involved fractional-pixel CO_2_ laser treatments, with OCT scans taken before the laser treatments, while the fourth visit only involved OCT scanning. Some of the participants also underwent additional OCT scanning 5 min after the laser treatment. The timeline is illustrated in Fig. 7.

During the OCT imaging session, four distinct vaginal locations were imaged: distal anterior, distal posterior, proximal anterior, and proximal posterior. The fractional-pixel CO_2_ laser system (FemiLift^TM^, Alma Lasers, Israel) was employed with a probe, which was carefully inserted up to the top of the vagina and gradually drawn out at 1-cm intervals while being rotated, providing complete circumferential treatment of the vagina. The laser beam is divided into 81 microbeams (pixels) at each activation (per 1 cm^2^), and the laser intensity was tunable dependent on the patient's tolerance, ranging from 40 to 120 mJ/pixel. In this study, the setting was 50–100 mJ/pixel, depending on each patient's comfort level. The laser treatment procedure was performed twice at each session. Prior to initiating vaginal laser therapy, a local anesthetic cream was applied to the introitus for 10 min, followed by thorough cleaning and drying.

### Statistical analysis

To evaluate the efficacy of the treatment and its impact on the vaginal tissue, we performed a comprehensive statistical analysis. The mean BVD and VET were calculated for each of the four locations from every visit. These metrics were then used to assess the changes in the microvasculature and epithelial thickness over time.

To further investigate the relationships between the treatment outcomes and the patients' clinical assessments, Pearson's correlation coefficients were computed for BVD, VHI, and VSQ. The strength of the correlation was interpreted as follows: an absolute value of the correlation coefficient between 0.1 and 0.3 indicated a weak correlation between variables; between 0.3 and 0.5, a moderate correlation; and greater than 0.5, a strong correlation.^29^

In addition to Pearson's correlation analysis, we explored other potential relationships among the variables, and we assessed the statistical significance of our findings using appropriate hypothesis tests. All statistical analyses were conducted using MATLAB and R, and the results were interpreted considering a significance level of 0.05.

## Data Availability

The data that support the findings of this study are available from the corresponding author upon reasonable request.
